# Growth and Phenology of Three Dwarf Shrub Species in a Six-Year Soil Warming Experiment at the Alpine Treeline

**DOI:** 10.1371/journal.pone.0100577

**Published:** 2014-06-23

**Authors:** Alba Anadon-Rosell, Christian Rixen, Paolo Cherubini, Sonja Wipf, Frank Hagedorn, Melissa A. Dawes

**Affiliations:** 1 Department of Plant Biology, University of Barcelona, Barcelona, Catalonia, Spain; 2 WSL Institute for Snow and Avalanche Research SLF, Davos, Switzerland; 3 Swiss Federal Institute for Forest, Snow and Landscape Research (WSL), Birmensdorf, Switzerland; National University of Mongolia, Mongolia

## Abstract

Global warming can have substantial impacts on the phenological and growth patterns of alpine and Arctic species, resulting in shifts in plant community composition and ecosystem dynamics. We evaluated the effects of a six-year experimental soil warming treatment (+4°C, 2007–2012) on the phenology and growth of three co-dominant dwarf shrub species growing in the understory of *Larix decidua* and *Pinus uncinata* at treeline in the Swiss Alps. We monitored vegetative and reproductive phenology of *Vaccinium myrtillus*, *Vaccinium gaultherioides* and *Empetrum hermaphroditum* throughout the early growing season of 2012 and, following a major harvest at peak season, we measured the biomass of above-ground ramet fractions. For all six years of soil warming we measured annual shoot growth of the three species and analyzed ramet age and xylem ring width of *V. myrtillus*. Our results show that phenology of the three species was more influenced by snowmelt timing, and also by plot tree species (*Larix* or *Pinus*) in the case of *V. myrtillus*, than by soil warming. However, the warming treatment led to increased *V. myrtillus* total above-ground ramet biomass (+36% in 2012), especially new shoot biomass (+63% in 2012), as well as increased new shoot increment length and xylem ring width (+22% and +41%, respectively; average for 2007–2012). These results indicate enhanced overall growth of *V. myrtillus* under soil warming that was sustained over six years and was not caused by an extended growing period in early summer. In contrast, *E. hermaphroditum* only showed a positive shoot growth response to warming in 2011 (+21%), and *V. gaultherioides* showed no significant growth response. Our results indicate that *V. myrtillus* might have a competitive advantage over the less responsive co-occurring dwarf shrub species under future global warming.

## Introduction

Alpine and Arctic ecosystems are predicted to be especially vulnerable to global warming [1], [2] because plant growth and performance in these environments are strongly constrained by low temperature, short growing seasons and frequent freezing events during the snow-free period. Projected increases of 1.8–4°C in the global mean surface air temperature by the end of the 21st century [3] can cause dramatic changes in community composition since co-occurring species respond differently to climate variations [4]–[6]. Community changes are of particular importance in alpine and Arctic ecosystems due to their potential effects on climate feedbacks through shifts in plant community composition and plant species cover [7], [8].

Experimental studies have shown that responses of plants growing at high latitude and high elevation to warming are species-specific [9]–[11], demonstrating that understanding how plant community dynamics might change with higher temperatures can only be achieved through assessing responses of individual species. For some species and study sites, positive growth responses to warming were transient, stopping some years after the experiment started [12], [13]. In contrast, other studies found a lag in plant growth response to experimental warming or inconsistent responses over time [6], [14]. Therefore, species-specific studies lasting several years are crucial for understanding community responses to global warming.

Phenological timing is an important factor for plant fitness and abundance, and it can be highly responsive to temperature ([15] and references therein). For instance, advanced leaf phenology in response to warming could lead to an extension of the photosynthetically active season, which, in turn, could lead to greater carbon gain and ultimately growth for plants [16], [17]. Advanced phenology of high-latitude and high-elevation species has occurred with experimentally increased growing season temperatures in numerous studies where snowmelt date was not modified [10], [18]–[22] (but see [23]). In a meta-analysis of alpine and Arctic studies, Arft et al. [12] suggested a relationship between advanced leaf bud burst under warming and increased vegetative growth in alpine and Arctic species. However, most studies have focused on phenology and growth separately, whereas the relationship between phenology and growth in response to warming at an individual/ramet level has rarely been reported.

Shrubs are an essential component of alpine and Arctic ecosystems, and several studies have reported an increase in their cover due to climate warming [6], [14], [24]–[27]. This “shrubification” strongly impacts ecosystem dynamics because shrubs modify patterns of snow accumulation, decrease albedo and modify carbon storage and nutrient cycling by greater biomass accumulation and more recalcitrant litter (see review by Myers-Smith et al. [8]).

Shrub growth responses to global warming in alpine and Arctic ecosystems have been assessed in many studies through long-term observations [5], [28], experimental manipulations [6] and/or studies using gradients across elevation or latitude [29], [30]. Most previous warming experiments have measured above-ground primary production by means of new shoot growth or standing biomass [11], [21], [31]–[33]. In contrast, relatively few studies have focused on shrub secondary growth [13], [34], [35] since acquiring these data requires destructive techniques. Therefore, it remains unclear if the reported increases in biomass production or in shoot increment length are caused by shifts in biomass allocation or if greater overall biomass production occurs.

The Stillberg treeline research area in the Swiss Central Alps hosted a six-year soil warming experiment (2007–2012). A short-term investigation of the dominant ericaceous dwarf shrub species growing in the understory of treeline trees after three years of warming showed that *Vaccinium myrtillus* responded to higher temperatures with increased shoot increment length but that *Vaccinium gaultherioides* and *Empetrum hermaphroditum* did not show a growth response [11]. Moreover, there were only few indications for changes in early-season vegetative phenology in these species under warming [36], [37]. In the study presented here, we conducted a detailed investigation of dwarf shrub phenological and growth responses during the summer of 2012, the final (sixth) year of the soil warming experiment. A final destructive harvest conducted at the peak of the vegetation period allowed us to investigate warming effects on secondary growth and biomass allocation for the first time. Our aims were (i) to determine whether a positive growth response in *Vaccinium myrtillus* was maintained after six years of soil warming, and whether *Vaccinium gaultherioides* and *Empetrum hermaphroditum* showed any delayed increases in growth after this full experimental period. By tracking the vegetative and flowering phenology of these dwarf shrubs, we also aimed (ii) to relate possible lagged changes in their phenology to changes in growth. Finally, we analyzed the widths of *V. myrtillus* growth rings with the aim of (iii) assessing whether the increases in shoot increment length under soil warming previously reported for this species represented an increase in the entire above-ground biomass of the ramets or if there was simply a shift in biomass allocation.

## Material and Methods

### Study site

The study site was located within the Stillberg treeline site in the Central Alps (Davos, Switzerland, 9° 52′E, 46° 46′ N). Stillberg is a 5-ha long-term afforestation research area where tree seedlings were planted into the intact dwarf shrub community in 1975 by the Swiss Federal Institute for Forest, Snow and Landscape Research (WSL). Climate data measured by a WSL meteorological station located within the research area (2090 m a.s.l.) indicated a mean annual precipitation of 1155 mm and mean annual air temperature of 2.1°C from 1975 to 2012. For the same period, the main growing season months (June-August) had a mean precipitation of 444 mm and a mean air temperature of 9.2°C. See Table S1 for details on climate data over the study years (2007–2012).

No specific permits were required for this location and activities and the field studies did not involve endangered or protected species.

### Experimental design

The experiment consisted of 40 hexagonal 1.1 m^2^ plots, 20 with one *Pinus mugo* ssp. *uncinata* (DC.) Domin individual in the center and 20 with one *Larix decidua* Mill. individual in the center. The plots were situated within an area of 2500 m^2^ on a NE-exposed 25–30° steep slope at 2180 m a.s.l. at or slightly above the current treeline in the region [38], [39]. The trees were sparsely distributed without forming a closed canopy; therefore, each plot contained a single tree surrounded by a dense cover of understory vegetation consisting mainly of the co-dominant dwarf shrub species *Vaccinium myrtillus* L., *Vaccinium gaultherioides* Bigelow (group *V. uliginosum* agg.) and *Empetrum nigrum* subsp. *hermaphroditum* (Hagerup) Böcher (referred to hereafter as *Empetrum hermaphroditum*). Further details about understory species composition were reported by Martin et al. [36] and Dawes et al. [11].

A free air CO_2_ enrichment (FACE) experiment was started after snowmelt in early June 2001 and applied during each snow-free season for nine years (ending in 2009). The 40 plots were assigned to 10 groups of four neighboring plots (two *Pinus* and two *Larix* trees per group) and half of these groups were randomly assigned to an elevated CO_2_ treatment while the other half served as controls (see a detailed description of the setup and performance of the CO_2_ enrichment facility in Hättenschwiler et al. [38] and in Dawes et al. [40]). Dwarf shrub responses to the CO_2_ enrichment have been reported in detail by Dawes et al. [11], [41]. A soil warming treatment was added to the experiment in spring 2007 and was applied during each snow-free season until early August 2012. Within each of the 10 CO_2_ treatment groups, one plot of each tree species was randomly selected and assigned a soil warming treatment, either control or warmed, and the second plot was assigned the other treatment, yielding a balanced split-split-plot design with a replication of five individual plots for each combination of CO_2_ level, soil warming treatment and tree species. Therefore, from 2007 to 2009 the experiment included both CO_2_ enrichment and soil warming, whereas from 2010 to 2012 the treatment consisted of soil warming only. The soil warming treatment was applied using 420 W-heating cables laid on the ground surface underneath the dwarf shrub layer, with a 5 cm distance between neighboring cables [42]. Each year, the heating was turned on immediately after snowmelt and turned off just before the site was covered in snow for the winter to avoid an interaction between soil temperature and snow cover duration. The warming treatment increased the growing season mean soil temperatures at 5 cm depth by 3.1 to 4.4°C over the 6 seasons of heating, with a difference of +3.5°C in 2012 (HOBO U23 Pro v2 dataloggers, Onset Computer Corporation, Bourne, MA, USA). Air temperature was warmed within the dwarf shrub canopy (0.9°C at 20 cm above ground [42]). The warming treatment had a slight drying effect on the soil organic layer (details in Hagedorn et al. [42] and Dawes et al. [11]). However, the soil matric water potential at 5 cm depth was always above -300 hPa in all plots, indicating overall very moist soil conditions [11], [43].

Snowmelt date of each plot from 2007 to 2011 was defined as the date in spring when soil temperatures at 5 cm depth rose sharply from values near 0°C, supported by visual estimations in the field and photographs. In 2012, snowmelt date was determined by visual estimations as the date when 50% of the plot was snow free and the ramets selected for detailed investigation were uncovered. This way, the estimation was more accurate for the study of the specific ramets selected.

### Phenology

At the time of snowmelt in 2012, we marked five ramets of *V. myrtillus*, *V. gaultherioides* and *E. hermaphroditum* in each plot, excluding the area within 10 cm of plot borders to avoid potential edge effects. All plant measurements were made on these ramets. *Vaccinium myrtillus* was present and abundant in every plot, *V. gaultherioides* was present in 35 of the 40 plots, and *E. hermaphroditum* was present in 26 of the 40 plots, which was sufficient replication to assess treatment effects.

We monitored the phenology of the three study species between the start of snowmelt (day of year 142) and peak growing season (day of year 212). We visited all plots and monitored all ramets every 2 to 4 days at early growing season and every 4 to 6 days after flowering. For each marked ramet, we recorded the date when it entered the following phenophases (some of which occurred at the same time): (1) burst of first vegetative bud, (2) first leaf starting to unfold, (3) first leaf fully expanded, (4) start of shoot elongation, (5) burst of first flower bud, (6) anthesis, and (7) first flower withered. In early August, we harvested all vegetation from the experimental plots. Previous phenological data in the same plots from an entire snow-free season [44] confirm our observation that dwarf shrub vegetative development was completed at the time of the harvest, whereas fruits were not mature and leaf senescence had not started.

### Shoot increment length and above-ground biomass

To track shoot growth responses during the whole soil warming experimental period, shoot increment length of the three species was measured from 2005 to 2011 on the longest branch of five to seven ramets in the field (2005–2009 data presented in Dawes et al. [11]). During early August 2012, we harvested all the marked ramets in every plot, clipping them at ground level. For *E. hermaphroditum*, we measured the new shoot increment length on the longest branch for every ramet before detaching and drying them for biomass measures. For all three species, we separated new shoots and leaves from the rest of the ramet while they were still fresh and dried them at 60°C for 48 h. For the two deciduous *Vaccinium* species, we weighed new shoots and leaves separately and measured the length of three new shoots per ramet, whereas for *E. hermaphroditum*, we weighed the small needle-like leaves and new shoots together. For *V. myrtillus* and *V. gaultherioides*, we counted the number of new shoots per ramet and calculated the average mass per individual shoot. For *E. hermaphroditum*, we counted the number of new shoots from a subsample of ramets and used the mass-count relationship to estimate the number of branches for the remaining ramets in each plot. For all three species, we dried the remaining ramet material (designated as “main stems”, >1 year old) at 60°C for 24 h and weighed it.

### Growth rings of Vaccinium myrtillus

We made cross-sections of 20 µm thickness from the basal 1.5 cm of dried *V. myrtillus* stems using a sledge microtome (WSL-Lab-microtome). We stained sections with a mixture of Safranin and Astrablue to emphasize the growth ring structure. For dehydrating the sections for preservation, we rinsed them with increasingly concentrated ethanol solutions (75%, 96%, 100%), immersed them in Xylol, imbedded them in Canada-Balsam and dried them at 60°C for 24 hours [45] before photographing them at x20–x200 magnification through a microscope with a digital camera (Canon EOS 650D on Olympus BX41 microscope; Fig. S1). We used the images to visually count rings and to measure xylem ring widths in four radii per section with the program WinCELL [46]. We excluded 23 out of 200 ramets from the statistical analyses because wood was damaged or broken or rings were not distinguishable. Ramets were not old enough for rings to be analyzed statistically using specialized dendrochronological software, so we visually cross-dated the samples to find possible missing rings. Latewood had not formed in the 2012 growth rings, indicating that secondary growth was not finished when we harvested the ramets.

### Statistical analysis

We applied linear mixed effects models fitted with the restricted maximum likelihood estimation method (REML) to assess treatment effects on phenology and growth parameters. We used likelihood ratio tests to determine whether the previous CO_2_ treatment and interactions between CO_2_ and the other treatments contributed significantly to the model fits as fixed effects. Previous investigations already reported a lack of soil warming x CO_2_ interactive effects [11], [41] and we did not find significant persistent effects of the CO_2_ enrichment; therefore, we pooled across CO_2_ treatments for all final analyses. Statistical models for phenology and biomass of the study species included warming treatment, plot tree species and their interaction as fixed effects. We included snowmelt date as a covariate when it contributed significantly to the model fits, i.e. in tests of the effect of soil warming and plot tree species on phenology.

Shoot increment length and growth ring width were analyzed as repeated measures and included treatment year (categorical variable) and all interactions between year, warming and plot tree species as additional fixed effects (after Dawes et al. [11]). Measurements averaged over 2005 and 2006 were included as a covariate to account for differences in shoot increment length or ring width before the warming treatment started. When a significant interaction between soil warming and year was found, we additionally tested the effect of soil warming with separate analyses for each individual year. We accounted for violation of independence of residuals from different treatment years by implementing the residual autocorrelation structure corAR1 [47]. As growth rings were not completely finished in 2012, we excluded this year from the repeated measures analysis of growth ring widths. We tested for Pearson's correlations between shoot increment length and above-ground biomass, as well as between shoot increment length and xylem ring width in 2012. We did not extend this analysis to previous years because ramets harvested in 2012 were not the same as those randomly selected for shoot increment measurements in previous years.

For all statistical analyses, the random effects structure reflected the hierarchy of the split-split-plot experimental design, with measurements made on ramets in 40 individual plots, nested within 20 soil warming treatment groups, nested within 10 CO_2_ treatment groups. For all analyses, we visually checked assumptions of normality and homoscedasticity of the residuals. We log-transformed response variables when necessary to reach these assumptions. Effects were considered significant at *P<*0.05 and, because of the relatively low replication, we considered *P*≥0.05 but <0.10 as marginally significant. We performed all the analyses with R version 2.15.2 [48] using the nlme package [47].

## Results

### Abiotic conditions

In 2012, snow melted from the experimental plots between 21 and 31 May (days of year 142 and 152). There were no differences in the date of snowmelt between soil warming treatments (*F_1,9_* = 0.64, *P* = 0.447) or plot tree species (*F_1,18_* = 1.04, *P* = 0.321). From 21 May to 3 August, when plants were harvested, the mean air temperature at the meteorological station was 9.7°C and temperatures ranged from -0.4 on 12 June to 21.9°C on 30 June.

### Phenology

**Figure 1:**
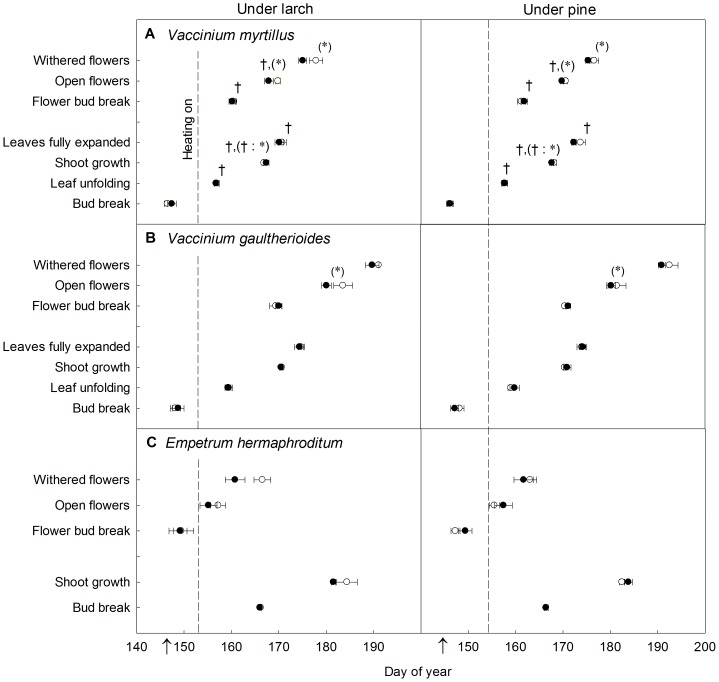
Vegetative and flowering phenology for the three dwarf shrub species studied. Vegetative and flowering phenology for the three dwarf shrub species studied. Circles represent the mean day of year (±1 SE) of each phenophase for the four different soil warming (open circles, unwarmed; closed circles, warmed) and plot tree species (larch or pine) combinations (n = 10). Crosses show significant differences between plot tree species (*P*<0.05) and marginally significant differences (0.05≤*P*<0.10) between soil warming treatments are given by asterisks in parentheses. Colons show treatment interactions. Note that in some cases symbols for different soil warming treatments overlap completely. Dotted lines show the date when the warming treatment started in 2012 and arrows on the x-axes show the mean snowmelt date for all plots with the same tree species.

The soil warming treatment did not significantly affect the vegetative phenology of the three dwarf shrub species (Fig. 1). However, we found marginally significant warming effects on their flowering phenology. *Vaccinium myrtillus* and *V. gaultherioides* showed a slightly earlier flower anthesis (by 1 and 2 days, respectively) in warmed plots than in unwarmed plots (*V. myrtillus*: *F_1,9_* = 4.3, *P* = 0.067, Fig.1A; *V. gaultherioides*: *F_1,7_* = 4.41, *P* = 0.074, Fig. 1B). *Vaccinium myrtillus* flowers also withered slightly earlier with increased soil temperatures (by 2 days, *F_1,9_* = 4.03, *P* = 0.076).

The tree species present in the plot had a greater effect than soil warming on *V. myrtillus* vegetative and reproductive phenology (Fig. 1A). The start of leaf unfolding, start of shoot elongation, flower bud break and flower anthesis took place 1 day earlier under larch than under pine (*P*<0.011), and leaf full expansion occurred 3 days earlier (*F_1,17_* = 29.88, *P*<0.001). There was a marginally significant effect of the tree species x warming interaction on the start of shoot elongation for *V. myrtillus*, which was slightly earlier (1 day) in warmed plots under larch than in unwarmed plots under pine (*F*_1,17_ = 3.7, *P* = 0.071). The phenology of *V. gaultherioides* and *E. hermaphroditum* did not show significant differences between plots with the two different tree species (Fig. 1B,C).

Most of the phenological phases for *V. myrtillus* and *V. gaultherioides* occurred earlier in plots with an earlier snowmelt date (*P*<0.07). However, *V. myrtillus* anthesis and *V. gaultherioides* flower bud break and withering did not show a relationship with snowmelt timing. *Empetrum hermaphroditum* flowering phenophases and start of shoot elongation were also related to snowmelt date (*P*<0.04), but vegetative bud break was not.

### Shoot increment length

**Figure 2:**
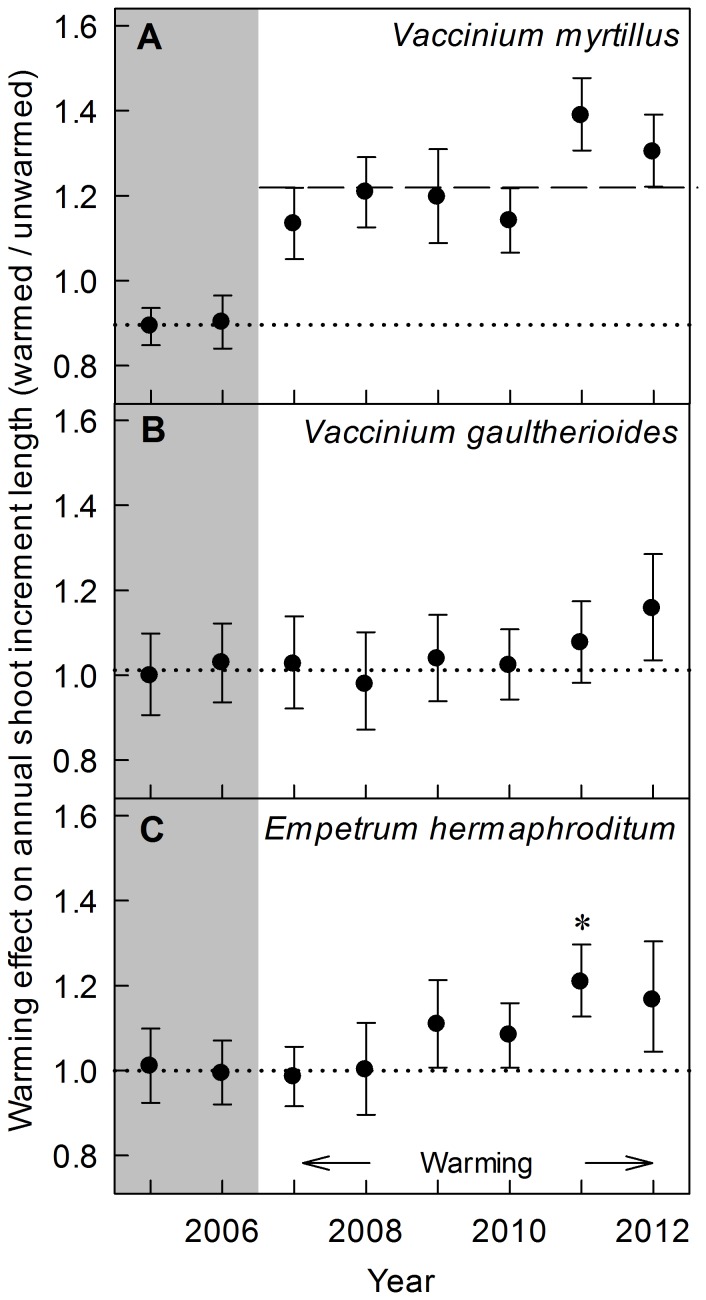
Soil warming effect on dwarf shrub annual shoot increment length. Soil warming effect on dwarf shrub annual shoot increment length from 2007 until 2012, the entire duration of the soil warming experiment. Data through 2009 were presented in Dawes et al. (2011a). The warming effect was calculated as the ratio of the mean shoot increment length of all warmed plots to the mean of all unwarmed plots, pooled across plots containing a larch or pine tree. Error bars represent ±1 SE of the ratio. The dashed line shows the significant warming effect on *V. myrtillus* averaged for 2007–2012. The asterisk shows significant differences between temperature treatments (*P*<0.05). Pre-warming ratios are shown in the shaded region (2005–2006) and the dotted line is drawn through the average of these two points, which indicates the mean warmed to unwarmed ratio before treatment began.

Soil warming had a significant positive effect on the annual shoot increment length of *V. myrtillus* (mean increase of 22% over 2007–2012, *F_1,9_* = 41.38, *P*<0.001, Fig. 2A). Treatment year also had a significant effect (*F*_5,180_ = 3.67, *P* = 0.004) but the warming x year effect was not significant (*F*_5,180_ = 1.89, *P* = 0.100). Nevertheless, the response was larger in the two last study years (shoot increment length 34% greater in warmed plots than unwarmed plots for 2011–2012) than in the previous years (18% greater in warmed plots than unwarmed plots, averaged over 2008–2010).

The annual shoot increment length of *V. gaultherioides* was not significantly affected by soil warming (*F_1,9_* = 0.99, *P* = 0.346, Fig. 2B) although there was a trend of increase with warming in the last year. Treatment year had a significant effect on *V. gaultherioides* shoot increment length (*F_5,171_* = 6.93, *P*<0.001) but the warming x year interaction was not significant (*F_5,171_* = 0.5, *P* = 0.776). *Empetrum hermaphroditum* annual shoot increment length showed a significant effect of treatment year (*F_5,128_* = 25.97, *P*<0.001, Fig. 2C) and the plot tree species x year interaction (*F_5,128_* = 2.55, *P* = 0.031), and also a marginally significant effect of warming (*F_1,9_* = 4.83, *P* = 0.056) and the warming x year interaction (*F_5,128_* = 2.21, *P* = 0.057). Analyses of individual years showed that *E. hermaphroditum* only had a significant (positive) shoot growth response to soil warming in 2011 (+21% increase in warmed plots compared to unwarmed plots, *F_1,7_* = 10.01, *P* = 0.016) and that the shoot increment length of this species was greater under pine than under larch in 2008 (marginally significant, *F_1,11_* = 4.04, *P* = 0.070) and 2009 (*F_1,10_* = 5.07, *P* = 0.048).

Shoot increment length averaged over 2005 and 2006 (pre-treatment covariate) positively influenced the length of new *V. gaultheroides* shoots during the 2007–2012 warming period (*F_1,17_* = 18.61, *P*<0.001), and to a lesser extent that of *V. myrtillus* and *E. hermaphroditum* (both marginally significant, *F_1,17_* = 3.28, *P* = 0.088 and *F_1,13_* = 4.58, *P* = 0.052, respectively). In 2012, new shoot increment length showed a positive correlation with new shoot biomass for each of the three species (*V. myrtillus R^2^* = 0.53, *P*<0.001; *V. gaultherioides R^2^* = 0.39, *P*<0.001; *E. hermaphroditum R^2^* = 0.27, *P*<0.001).

### Above-ground biomass

**Figure 3:**
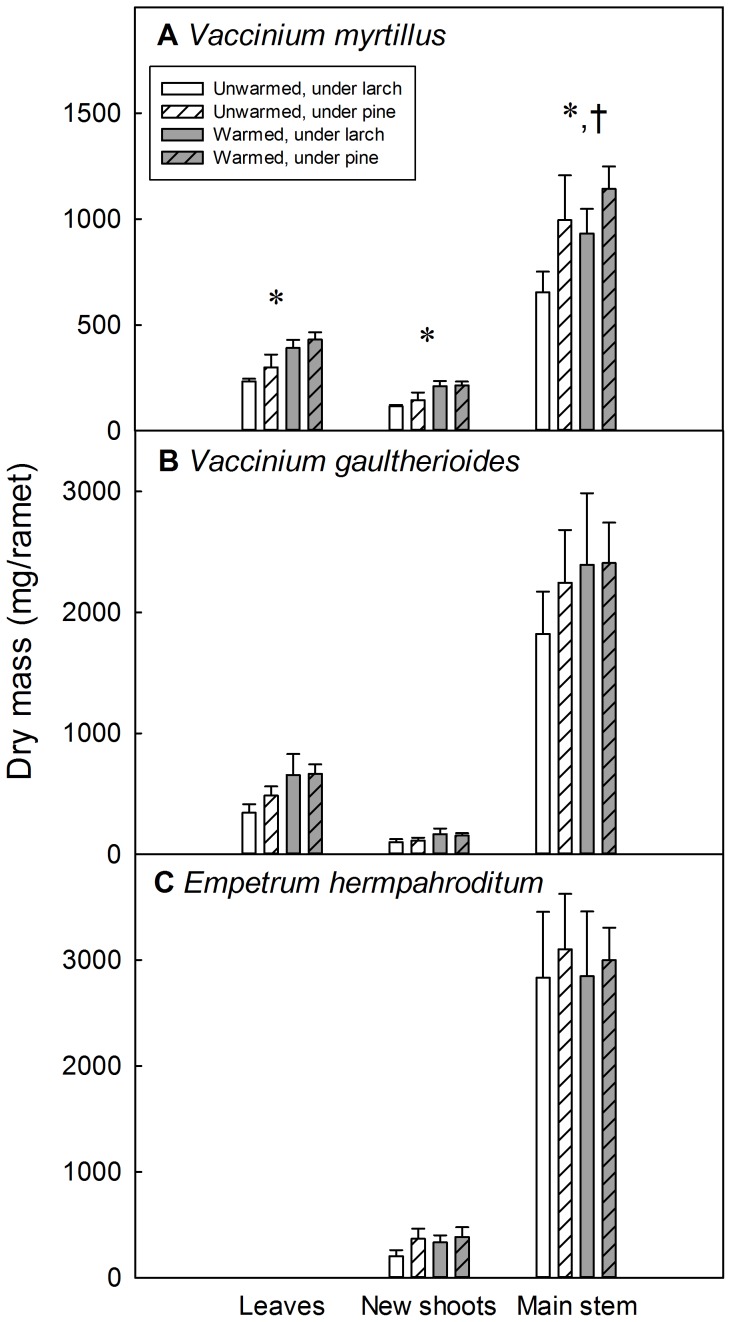
Above-ground biomass partitioning of the three dwarf shrub species studied. Above-ground biomass partitioning of the study species for each soil warming and plot trees species combination (mean values +1 SE, n = 10). Asterisks show significant differences between soil warming treatments and crosses show significant differences between plot tree species (*P*<0.05). For *Empetrum hermaphroditum* only, leaves and new shoots are both included in “New shoots”. The y-axis scale varies across species to emphasize differences between treatments.

At the time of harvest in August 2012, *V. myrtillus* ramets showed a 54% greater total leaf biomass (*F_1,9_* = 15.52, *P* = 0.003), a 63% greater total new shoot biomass (*F_1,9_* = 14.15, *P* = 0.005) and a 26% greater main stem (stems >1 year old) biomass (*F_1,9_* = 7.82, *P* = 0.021) in warmed plots than in unwarmed plots (Fig. 3A). Moreover, the main stem biomass of *V. myrtillus* was 35% greater under pine than under larch (*F_1,18_* = 5.15, *P* = 0.036). Although differences in ramet above-ground biomass between warming treatments were, on average, larger in plots with larch than in plots with pine, there were no significant tree species x warming interactions for any of the plant parts (*P*>0.77). *Vaccinium gaultherioides* and *E. hermaphroditum* did not show significant differences among treatments in the above-ground biomass for any of the plant parts analyzed (Fig. 3B,C). The three dwarf shrub species showed a positive correlation between new shoot biomass and total ramet biomass (*V. myrtillus R^2^* = 0.77, *P*<0.001; *V. gaultherioides R^2^* = 0.71, *P* < 0.001; *E. hermaphroditum R^2^* = 0.52, *P*<0.001).

The number of new shoots per ramet in *V. myrtillus* was significantly greater in warmed plots than in unwarmed plots (*F_1,9_* = 6.466, *P* = 0.032) and slightly greater under pine than under larch (marginally significant, *F_1,18_* = 3.51, *P* = 0.077). In addition, the mass per individual shoot was larger in warmed plots than in unwarmed plots (*F_1,9_* = 9.79, *P* = 0.012) and slightly larger under larch than under pine (marginally significant, *F_1,18_* = 4.14, *P* = 0.060). Therefore, *V. myrtillus* not only produced more shoots in warmed plots, but these individual shoots were longer and heavier. *Vaccinium gaultherioides* produced slightly more new shoots in warmed plots than in unwarmed plots (marginally significant, *F_1,9_* = 3.74, *P* = 0.085) and under pine than under larch (*F_1,13_* = 5.91, *P* = 0.030), but the mass per individual shoot did not differ between warming treatments or plot tree species.

### Growth rings of Vaccinium myrtillus

We did not find significant differences in ramet age between warming treatments (8.4±0.5 years old for unwarmed and 7.9±0.3 for warmed plots; *F_1,9_* = 0.58, *P* = 0.467; Fig. S2). However, ramets were younger under larch (7.4±0.4 years old) than under pine (9.0±0.4; *F_1,18_* = 10.13, *P* = 0.005). There was a marginally significant warming x tree species interaction (*F_1,18_* = 3.28, *P* = 0.087): on average, ramets were older in unwarmed plots with pine than in both warmed and unwarmed plots with larch.

**Figure 4:**
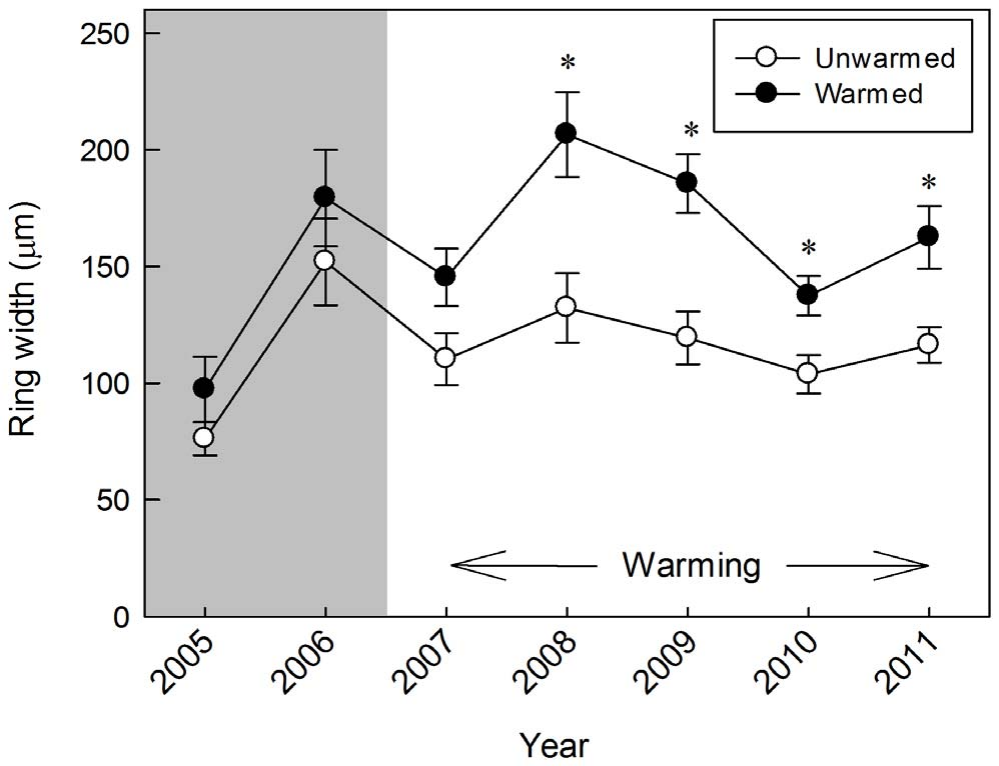
Xylem ring widths of *Vaccinium myrtillus*. Xylem ring widths of *Vaccinium myrtillus* from 2007 to 2011 for warmed and unwarmed plots, pooled across plots containing a larch or pine tree (mean values ±1 SE, n = 20). Asterisks show significant differences between soil warming treatments (*P*<0.05). Pre-warming values are shown in the shaded region (2005–2006).

Repeated measures analyses showed that, averaged across all treatment years, *V. myrtillus* ring width was 41% greater in warmed plots than in unwarmed plots (*F*_1,9_ = 16.45, *P* = 0.003, Fig. 4). Treatment year and the warming x year interaction also had significant effects (*F_4,297_* = 4.67, *P* = 0.001 and *F_4,297_* = 3.32, *P* = 0.011, respectively), as well as the pre-treatment ring width covariate (*F*_1,44_ = 17.26, *P*<0.001). Plot tree species and interactions between tree species and the other fixed effects did not significantly influence xylem ring width (*P*>0.17). Analyses of individual years showed that warming had a significant effect on ring width in all years (*P*<0.024) except for the first year of treatment (Fig. 4).

Although xylem latewood formation was not completed when we collected the samples in 2012, we found a significant correlation between shoot increment length and ring width in that year (*R^2^* = 0.157, *P*<0.001), as well as between ring width and biomass of new shoots (*R^2^* = 0.088, P<0.001) and biomass of leaves (*R^2^* = 0.068, P<0.001). We tested for an age-biomass correlation and results corroborated that older ramets were heavier (*R^2^* = 0.24, *P*<0.001).

## Discussion

### Increased above-ground growth after six years of soil warming

Six years of soil warming led to a sustained growth stimulation of *V. myrtillus*, and the greatest response of new shoot increments occurred in the final two years. This increasing response over time contrasts with studies where positive responses of *V. myrtillus* and other alpine and Arctic plant species to warming were transient and stopped after four or five years [12], [13], [49]. In addition, our findings indicate that other factors that can potentially limit growth, such as nutrient or light availability, did not constrain the warming response of *V. myrtillus* in later years of the experiment [12]. Earlier studies at the same experimental site found more severe freezing damage of *V. myrtillus* under warming than in control plots [36], [37], but our results show that these freezing effects were not large enough to substantially offset the enhanced growth over six years. The other two dwarf shrub species showed at least a slight trend of increased shoot increment length with soil warming in the last years of the study, suggesting that the response of these two species to climate warming may be considerably delayed.

The species-specific warming effects on above-ground biomass production in our experiment contrast with a two-year study with open-top polythene tents in subarctic Sweden, where all three study species in common with our study showed a greater above-ground biomass under warming [50]. A five-year study at the same site in the subarctic by Press et al. [51] similarly showed that higher temperatures increased *V. uliginosum* (comparable with *V. gaultherioides*) biomass. Moreover, *Empetrum nigrum* shoot production and shoot increment length increased under two years [31] and five years [32] of warming with open-top chambers in alpine heath in Japan. The different methodologies applied (air warming by open-top chambers *vs*. soil warming by heating cables) might explain some of the differences between studies. In addition, different plant community composition and dynamics might play an important role in explaining the different results across study sites.

*Vaccinium myrtillus* has a wider elevational distribution (colline to alpine) than *V. gaultherioides* and *E. hermaphroditum* (subalpine to alpine) [52]. Its presence in lower elevational areas indicates that *V. myrtillus* might be adapted to higher temperatures than *V. gaultherioides* and *E. hermaphroditum* [11] and thus may be a stronger competitor under increased temperatures. In fact, a 22-year experiment with open-top greenhouses in the Swedish tundra reported no effects on *E. hermaphroditum* growth under 4°C of air warming [13], whereas in the same experiment, Graglia et al. [53] found positive effects on this species after six years of 2.5°C warming but not 4°C warming. The mean daily air temperature increase with open-top chambers is around 1-2°C [54] whereas the soil temperature increase by our heating cables is around 4°C, which is similar to the air temperature increase reached by Campioli et al. [13]. As suggested in their study, *E. hermpahroditum* might suffer from heat stress under high temperatures, responding positively only to smaller increases.

### Phenology: effects of growing season temperature, snowmelt and plot tree species

The lack of vegetative phenological responses to soil warming of the three dwarf shrub species studied and the strong effect of snowmelt date on early-season phenology in 2012 are consistent with results from the first three years of the experiment [37]. Some plant species have been shown not to respond to spring warming experiments [55] because they either do not respond to climate warming [55], [56], because they are more sensitive to changes in late winter temperatures [55] or because they primarily respond to other cues such as photoperiod [57]. In our experiment, the lack of warming effects on the vegetative phenophases might be partially due to the fact that vegetative development, especially for the two *Vaccinium* species, started directly after snowmelt, which coincided with the start of the warming treatment. Phenophases occurring later in the season (i.e. flowering phenophases in *Vaccinium* spp.) did not depend on the snowmelt date and were slightly influenced by the warming treatment, which is consistent with patterns found for tundra dwarf shrub species in subarctic Alaska [58].

Our study might have underestimated phenological responses to future climate warming because only growing season temperatures were altered [55], [59], whereas late winter temperatures, which can be key determinants of plant phenology [15], [55], were not experimentally manipulated. Nevertheless, other studies that only altered growing season temperatures found that the same species as in our study showed phenological advances under air warming [18] or that other alpine species showed a lagged response after multiple years of warming [10]. The distinct methodologies applied in these experiments (e.g. soil *vs.* air warming), microclimate conditions, different plant community types and genetic variations between populations might explain these differences [11], [60], [61].

The tree species present in the plot had a greater effect on *V. myrtillus* phenology than soil warming. The phenological advancement under larch could be due to a greater light incidence under this species at the beginning of the growing season, which could act as a phenological cue [62].

### Increased growth in *V. myrtillus* decoupled from phenology

The increased growth and biomass production found in *V. myrtillus* with soil warming did not correspond to a phenological shift or to the date of snowmelt, which indicates that *V. myrtillus* can produce more biomass under higher growing season temperatures even without a springtime extension of the active season. The peak-season harvest meant that we could not check if *V. myrtillus* phenology at senescence time was delayed by the warming treatment, an effect that has been reported for tundra plants in experiments with open-top chambers [63], [64]. Delayed leaf senescence could have influenced *V. myrtillus* growth in the late season (e.g. secondary growth) or in the following year. However, monitoring of leaf senescence in 2008–2009 showed no differences between warming treatments in this species (Dawes, unpublished) and late-season phenophases of alpine plants are generally considered to be more controlled by photoperiod than by temperature [65].

As advanced phenology could not explain the enhanced growth of *V. myrtillus* under soil warming, potential mechanisms for this response include increased rates of photosynthesis [66] and tissue formation [65] directly caused by higher temperatures. Another mechanism could be accelerated decomposition and mineralization of soil organic matter under warmer soil, which can improve nutrient availability for plants in alpine and Arctic environments where low temperatures tend to constrain these processes [49], [66]–[68]. Soil inorganic nitrogen content increased during the first three years of our soil warming experiment [11], suggesting that indirect effects of soil warming via an enhanced nutrient cycling played a role in the *V. myrtillus* growth response.

### Above-ground biomass increase under soil warming

To our knowledge, this is a pioneer study on assessing the effects of experimental soil warming on the age and xylem ring width of *V. myrtillus*. Our results show that rings were wider in warmed plots since the second year of treatment, a response that was maintained throughout the six experimental years. In addition, the significant positive correlation between shoot increment length and early xylem growth (xylem latewood formation was not completed) indicates that vessel size in the early xylem growing season is tightly related to the elongation of new shoots, which receive water from newly-formed vessels [69].

Moreover, the positive correlation between shoot increment length and both early xylem growth and annual shoot biomass production, together with the greater biomass in *V. myrtillus* in warmed plots, provide evidence that the previously reported increases in the shoot increment length of *V. myrtillus* with warming were not merely a result of a shift in biomass allocation but an overall increase in above-ground biomass production. However, below-ground biomass was not measured in this study and thus, it is possible that soil warming led to shifts in biomass allocation between below-ground and above-ground compartments [70].

Although warmer temperatures led to an increased growth of *V. myrtillus*, the age structure of this species was not affected by soil warming. However, there was a lower ramet turnover under pine (older ramets) than under larch, and ramet main stem biomass was also higher under pine. A possible explanation for these differences is that pine provides greater protection against freezing conditions at the beginning of the season before needles are developed on deciduous larch. This effect would be similar to the facilitation exerted by shrubs on young trees [71], [72], leading to lower mortality rates. Moreover, Dawes et al. [11] found less canopy shading under pine than under larch, thus the lower main stem biomass under larch may be a consequence of lower light availability.

### Concluding remarks

In summary, we found increased growth of *V. myrtillus* under soil warming, a response that was sustained, and even became stronger in the case of shoot increment length, over six years of warming. The application of dendrochronological techniques showed that this increased growth reflected an overall increase in above-ground biomass production. Moreover, the lack of an advanced phenology of *V. myrtillus* under soil warming indicated that an extended growing period was not necessary for the observed growth response. Our results suggest that *V. myrtillus* will experience a larger and more rapid growth benefit from a warming climate than *V. gaultherioides* or *E. hermaphroditum* and could therefore become increasingly dominant in high-elevation treeline environments.

## Supporting Information

Figure S1 **Cross-section of a** ***Vaccinium myrtillus*** **ramet.**
(PDF)

Figure S2 **Age of** ***Vaccinium myrtillus*** **for each soil warming treatment and plot tree species combination.**
(PDF)

Table S1 **Climate conditions in the experimental plots during all study years (2007–2012).**
(PDF)

Dataset S1 **Phenology, above-ground biomass, shoot elongation and xylem growth rings measurements.**
(XLSX)

## References

[1] Chapin III FS, Mcguire AD, Randerson J, Pielke R, Baldocchi D, et al (2000) Arctic and boreal ecosystems of western North America as components of the climate system. Global Change Biol 6: 211–223. 10.1046/j.1365-2486.2000.06022.x 35026938

[2] IPCC (2007) Climate Change 2007: Synthesis Report. Contribution of Working Groups I, II and III to the Fourth Assessment Report of the Intergovernmental Panel on Climate Change, IPCC, Geneva, Switzerland, 73 p.

[3] Meehl GA, Stocker TF, Collins WD, Friedlingstein AT, Gaye JM, et al.. (2007) Global climate projections. In: Climate change 2007: the physical science basis. Contribution of working group I to the fourth assessment report of the intergovernmental panel on climate change (eds Solomon S, Qin D, Manning M, et al.). Cambridge University Press, Cambridge, UK. pp. 747–845.

[4] Cornelissen JHC, Callaghan TV, Alatalo JM, Michelsen A, Graglia E, et al (2001) Global change and arctic ecosystems: Is lichen decline a function of increases in vascular plant biomass? J Ecol 89: 984–994.

[5] Hudson JMG, Henry GHR (2009) Increased plant biomass in a high arctic heath community from 1981 to 2008. Ecology 90: 2657–2663. 19886474 10.1890/09-0102.1

[6] Elmendorf SC, Henry GHR, Hollister RD, Björk RG, Bjorkman AD, et al (2012a) Global assessment of experimental climate warming on tundra vegetation: Heterogeneity over space and time. Ecol Lett 15: 164–175. 22136670 10.1111/j.1461-0248.2011.01716.x

[7] Wookey PA, Aerts R, Bardgett RD, Baptist F, Bråthen KA, et al (2009) Ecosystem feedbacks and cascade processes: Understanding their role in the responses of Arctic and alpine ecosystems to environmental change. Global Change Biol 15: 1153–1172.

[8] Myers-Smith IH, Forbes BC, Wilmking M, Hallinger M, Lantz T, et al (2011) Shrub expansion in tundra ecosystems: Dynamics, impacts and research priorities. Environ Res Lett 6: 10.1088/1748–9326/6/4/045509.

[9] Klanderud K (2008) Species-specific responses of an alpine plant community under simulated environmental change. J Veg Sci 19: 363–372.

[10] Hoffmann AA, Camac JS, Williams RJ, Papst W, Jarrad FC, et al (2010) Phenological changes in six Australian subalpine plants in response to experimental warming and year-to-year variation. J Ecol 98: 927–937.

[11] Dawes MA, Hagedorn F, Zumbrunn T, Handa IT, Hättenschwiler S, et al (2011a) Growth and community responses of alpine dwarf shrubs to in situ CO_2_ enrichment and soil warming. New Phytol 191: 806–818. 21770945 10.1111/j.1469-8137.2011.03722.x

[12] Arft AM, Walker MD, Gurevitch J, Alatalo JM, Bret-Harte MS, et al (1999) Responses of tundra plants to experimental warming: Meta-analysis of the International Tundra Experiment. Ecol Monogr 69: 491–511.

[13] Campioli M, Leblans N, Michelsen A (2012) Twenty-two years of warming, fertilisation and shading of subarctic heath shrubs promote secondary growth and plasticity but not primary growth. PLoS ONE 7 , 10.1371/journal.pone.0034842. 10.1371/journal.pone.0034842 PMC3325270 22511968

[14] Chapin FS, Shaver GR, Giblin AE, Nadelhoffer KJ, Laundre JA (1995) Responses of Arctic tundra to experimental and observed changes in climate. Ecology 76: 694–711.

[15] Walther GR (2003) Plants in a warmer world. Perspect Plant Ecol 6: 169–185.

[16] Goulden ML, Munger JW, Fan S-M, Daube BC, Wofsy SC (1996) Exchange of carbon dioxide by a deciduous forest: Response to interannual climate variability. Science 271: 1576–1578.

[17] Myneni RB, Keeling CD, Tucker CJ, Asrar G, Nemani RR (1997) Increased plant growth in the northern high latitudes from 1981 to 1991. Nature 386: 698–702.

[18] Suzuki S, Kudo G (2000) Responses of alpine shrubs to simulated environmental change during three years in the mid-latitude mountain, northern Japan. Ecography 23: 553–564.

[19] Dunne JA, Harte J, Taylor KJ (2003) Subalpine meadow flowering phenology responses to climate change: Integrating experimental and gradient methods. Ecol Monogr 73: 69–86.

[20] Aerts R, Cornelissen JHC, Dorrepaal E, Van Logtestijn RSP, Callaghan TV (2004) Effects of experimentally imposed climate scenarios on flowering phenology and flower production of subarctic bog species. Global Change Biol 10: 1599–1609.

[21] Xu Z-F, Hu T-X, Wang K-Y, Zhang Y-B, Xian J-R (2009) Short-term responses of phenology, shoot growth and leaf traits of four alpine shrubs in a timberline ecotone to simulated global warming, Eastern Tibetan Plateau, China. Plant Spec Biol 24: 27–34.

[22] Smith J, Sconiers W, Spasojevic M, Ashton I, Suding K (2012) Phenological changes in alpine plants in response to increased snowpack, temperature, and nitrogen. Arct Antarct Alp Res 44: 135–142.

[23] Totland Ø, Alatalo JM (2002) Effects of temperature and date of snowmelt on growth, reproduction, and flowering phenology in the arctic/alpine herb, *Ranunculus glacialis* . Oecologia 133: 168–175. 28547303 10.1007/s00442-002-1028-z

[24] Tape K, Sturm M, Racine C (2006) The evidence for shrub expansion in Northern Alaska and the Pan-Arctic. Global Change Biol 12: 686–702.

[25] Rundqvist S, Hedenås H, Sandström A, Emanuelsson U, Eriksson H, et al (2011) Tree and shrub expansion over the past 34 years at the tree-line near Abisko, Sweden. Ambio 40: 683–692. 21954730 10.1007/s13280-011-0174-0 PMC3357856

[26] Ropars P, Boudreau S (2012) Shrub expansion at the forest-tundra ecotone: Spatial heterogeneity linked to local topography. Environ Res Lett 7: 10.1088/1748–9326/7/1/015501.

[27] Elmendorf SC, Henry GHR, Hollister RD, Björk RG, Boulanger-Lapointe N, et al (2012b) Plot-scale evidence of tundra vegetation change and links to recent summer warming. Nature Climate Change 2: 453–457.

[28] Hill GB, Henry GHR (2011) Responses of High Arctic wet sedge tundra to climate warming since 1980. Global Change Biol 17: 276–287.

[29] Rixen C, Schwoerer C, Wipf S (2010) Winter climate change at different temporal scales in *Vaccinium myrtillus*, an Arctic and alpine dwarf shrub. Polar Res 29: 85–94.

[30] Pato J, Ramón Obeso J (2012) Growth and reproductive performance in bilberry (*Vaccinium myrtillus*) along an elevation gradient. Ecoscience 19: 59–68.

[31] Wada N, Shimono M, Miyamoto M, Kojima S (2002) Warming effects on shoot developmental growth and biomass production in sympatric evergreen alpine dwarf shrubs *Empetrum nigrum* and *Loiseleuria procumbens* . Ecol Res 17: 125–132.

[32] Kudo G, Suzuki S (2003) Warming effects on growth, production, and vegetation structure of alpine shrubs: A five-year experiment in northern Japan. Oecologia 135: 280–287. 12698350 10.1007/s00442-003-1179-6

[33] Van Wijk MT, Clemmensen KE, Shaver GR, Williams M, Callaghan TV, et al (2004) Long-term ecosystem level experiments at Toolik Lake, Alaska, and at Abisko, Northern Sweden: Generalizations and differences in ecosystem and plant type responses to global change. Global Change Biol 10: 105–123.

[34] Bret-Harte MS, Shaver GR, Chapin III FS (2002) Primary and secondary stem growth in arctic shrubs: Implications for community response to environmental change. J Ecol 90: 251–267.

[35] Campioli M, Schmidt NM, Albert KR, Leblans N, Ro-Poulsen H, et al (2013) Does warming affect growth rate and biomass production of shrubs in the High Arctic? Plant Ecol 214: 1049–1058.

[36] Martin MA, Gavazov K, Körner C, Hättenschwiler S, Rixen C (2010) Reduced early growing season freezing resistance in alpine treeline plants under elevated atmospheric CO_2_ . Global Change Biol 16: 1057–1070.

[37] Rixen C, Dawes MA, Wipf S, Hagedorn F (2012) Evidence of enhanced freezing damage in treeline plants during six years of CO_2_ enrichment and soil warming. Oikos 121: 1532–1543.

[38] Hättenschwiler S, Handa IT, Egli L, Asshoff R, Ammann W, et al (2002) Atmospheric CO_2_ enrichment of alpine treeline conifers. New Phytol 156: 363–375. 10.1046/j.1469-8137.2002.00537.x 33873574

[39] Barbeito I, Dawes MA, Rixen C, Senn J, Bebi P (2012) Factors driving mortality and growth at treeline: a 30-year experiment of 92 000 conifers. Ecology 93: 389–401. 22624320 10.1890/11-0384.1

[40] Dawes MA, Hättenschwiler S, Bebi P, Hagedorn F, Handa IT, et al (2011b) Species-specific tree growth responses to 9 years of CO_2_ enrichment at the alpine treeline. J Ecol 99: 383–394.

[41] Dawes MA, Hagedorn F, Handa IT, Streit K, Ekblad A, et al (2013) An alpine treeline in a carbon dioxide-rich world: Synthesis of a nine-year free-air carbon dioxide enrichment study. Oecologia 171: 623–637. 23340765 10.1007/s00442-012-2576-5

[42] Hagedorn F, Martin MA, Rixen C, Rusch S, Bebi P, et al (2010) Short-term responses of ecosystem carbon fluxes to experimental soil warming at the Swiss alpine treeline. Biogeochemistry 97: 7–19.

[43] Dawes MA, Zweifel R, Dawes N, Rixen C, Hagedorn F (2014) CO_2_ enrichment alters diurnal stem radius fluctuations of 36-yr-old *Larix decidua* growing at the alpine tree line. New Phytol: 10.1111/nph.12742. 10.1111/nph.12742 24571288

[44] Zumbrunn T (2004) Alpine dwarf shrubs in a CO_2_ enriched world. Unpublished Diploma thesis, University of Basel, Basel, Switzerland.

[45] Schweingruber FH (1990) Microscopic Wood Anatomy. 3rd ed. Swiss Federal Institute for Forest, Snow and Landscape Research, Birmensdorf, 226 p.

[46] Régent Instruments, Inc. (2001) WinCELL pro 2001: image analysis software [computer program]. Québec.

[47] Pinheiro J, Bates D, DebRoy S, Sarkar D, The R Core Team (2008) NLME: linear and nonlinear mixed effects models. R package version 3: .1–89.

[48] R Core Team (2012) R: A language and environment for statistical computing. R Foundation for Statistical Computing, Vienna, Austria. ISBN 3-900051-07-0, URL http://www.R-project.org/

[49] Hartley AE, Neill C, Melillo JM, Crabtree R, Bowles FP (1999) Plant performance and soil nitrogen mineralization in response to simulated climate change in subarctic dwarf shrub heath. Oikos 86: 331–343.

[50] Parsons AN, Welker JM, Wookey PA, Press MC, Callaghan TV, et al (1994) Growth responses of four sub-Arctic dwarf shrubs to simulated environmental change. J Ecol 82: 307–318.

[51] Press MC, Potter JA, Burke MJW, Callaghan TV, Lee JA (1998) Responses of a subarctic dwarf shrub heath community to simulated environmental change. J Ecol 86: 315–327.

[52] Lauber K, Wagner G, Gygax A (2012) Flora Helvetica. Bern: Haupt. 1656 pp.

[53] Graglia E, Jonasson S, Michelsen A, Schmidt IK (1997) Effects of shading, nutrient application and warming on leaf growth and shoot densities of dwarf shrubs in two arctic-alpine plant communities. Ecoscience 4: 191–198.

[54] Marion GM, Henry GHR, Freckman DW, Johnstone J, Jones G, et al (1997) Open-top designs for manipulating field temperature in high-latitude ecosystems. Global Change Biol 3: 20–32.

[55] Cook BI, Wolkovich EM, Parmesan C (2012) Divergent responses to spring and winter warming drive community level flowering trends. P Natl Acad Sci USA 109: 9000–9005. 10.1073/pnas.1118364109 PMC3384199 22615406

[56] McEwan RW, Brecha RJ, Geiger DR, John GP (2011) Flowering phenology change and climate warming in southwestern Ohio. Plant Ecol 212: 55–61.

[57] Körner C, Basler D (2010) Phenology under global warming. Science 327: 1461–1462. 20299580 10.1126/science.1186473

[58] Wipf S (2010) Phenology, growth, and fecundity of eight subarctic tundra species in response to snowmelt manipulations. Plant Ecol 207: 53–66.

[59] Wolkovich EM, Cook BI, Allen JM, Crimmins TM, Betancourt JL, et al (2012) Warming experiments underpredict plant phenological responses to climate change. Nature 485: 494–497. 22622576 10.1038/nature11014

[60] Oberbauer SF, Elmendorf SC, Troxler TG, Hollister RD, Rocha AV, et al (2013) Phenological response of tundra plants to background climate variation tested using the International Tundra Experiment. Philos T Roy Soc B 368: 20120481. 10.1098/rstb.2012.0481 PMC3720054 23836787

[61] Schwartz MD, Hanes JM, Liang L (2013) Separating temperature from other factors in phenological measurements. Int J Biometeorol, 10.1007/s00484-013-0723-2. 10.1007/s00484-013-0723-2 23995622

[62] Pau S, Wolkovich EM, Cook BI, Davies TJ, Kraft NJB, et al (2011) Predicting phenology by integrating ecology, evolution and climate science. Global Change Biol 17: 3633–3643.

[63] Suzuki S, Kudo G (1997) Short-term effects of simulated environmental change on phenology, leaf traits, and shoot growth of alpine plants on a temperate mountain, northern Japan. Global Change Biol 3: 108–115.

[64] Natali SM, Schuur EAG, Rubin RL (2012) Increased plant productivity in Alaskan tundra as a result of experimental warming of soil and permafrost. J Ecol 100: 488–498.

[65] Körner C (2003) Alpine Plant Life: Functional Plant Ecology of High Mountain Ecosystems. 2nd ed. Springer, Berlin, 344 p.

[66] Rustad LE, Campbell JL, Marion GM, Norby RJ, Mitchell MJ, et al (2001) A meta-analysis of the response of soil respiration, net nitrogen mineralization, and aboveground plant growth to experimental ecosystem warming. Oecologia 126: 543–562. 28547240 10.1007/s004420000544

[67] Aerts R, Cornelissen JHC, Dorrepaal E (2006) Plant performance in a warmer world: General responses of plants from cold, northern biomes and the importance of winter and spring events. Plant Ecol 182: 65–77.

[68] Streit K, Hagedorn F, Hiltbrunner D, Portmann M, Saurer M, et al (2014) Soil warming alters microbial substrate use in alpine soils. Global Change Biol 20: 1327–1338. 10.1111/gcb.12396 24106016

[69] Esau K (1977) Anatomy of seed plants. 2nd ed. John Wiley & Sons, New York, 550 p.

[70] Atkin OK, Loveys BR, Atkinson LJ, Pons TL (2006) Phenotypic plasticity and growth temperature: understanding interspecific variability. J Exp Bot 57: 267–281. 16371402 10.1093/jxb/erj029

[71] Gómez-Aparicio L, Zamora R, Castro J, Hódar JA (2008) Facilitation of tree saplings by nurse plants: Microhabitat amelioration or protection against herbivores? J Veg Sci 19: 161–172.

[72] Grau O, Ninot JM, Blanco-Moreno JM, van Logtestijn RSP, Cornelissen JHC, et al (2012) Shrub-tree interactions and environmental changes drive treeline dynamics in the Subarctic. Oikos 121: 1680–1690.

