# Supplementary material for: Growth and Phenology of Three Dwarf Shrub Species in a Six-Year Soil Warming Experiment at the Alpine Treeline
Source: PLoS One. 2014 Jun 23;9(6):e100577. doi: 10.1371/journal.pone.0100577 (PMC4067323; doi:10.1371/journal.pone.0100577)
Supplement: Figure S1 — Cross-section of a Vaccinium myrtillus ramet. (PDF) [file pone.0100577.s001.pdf]

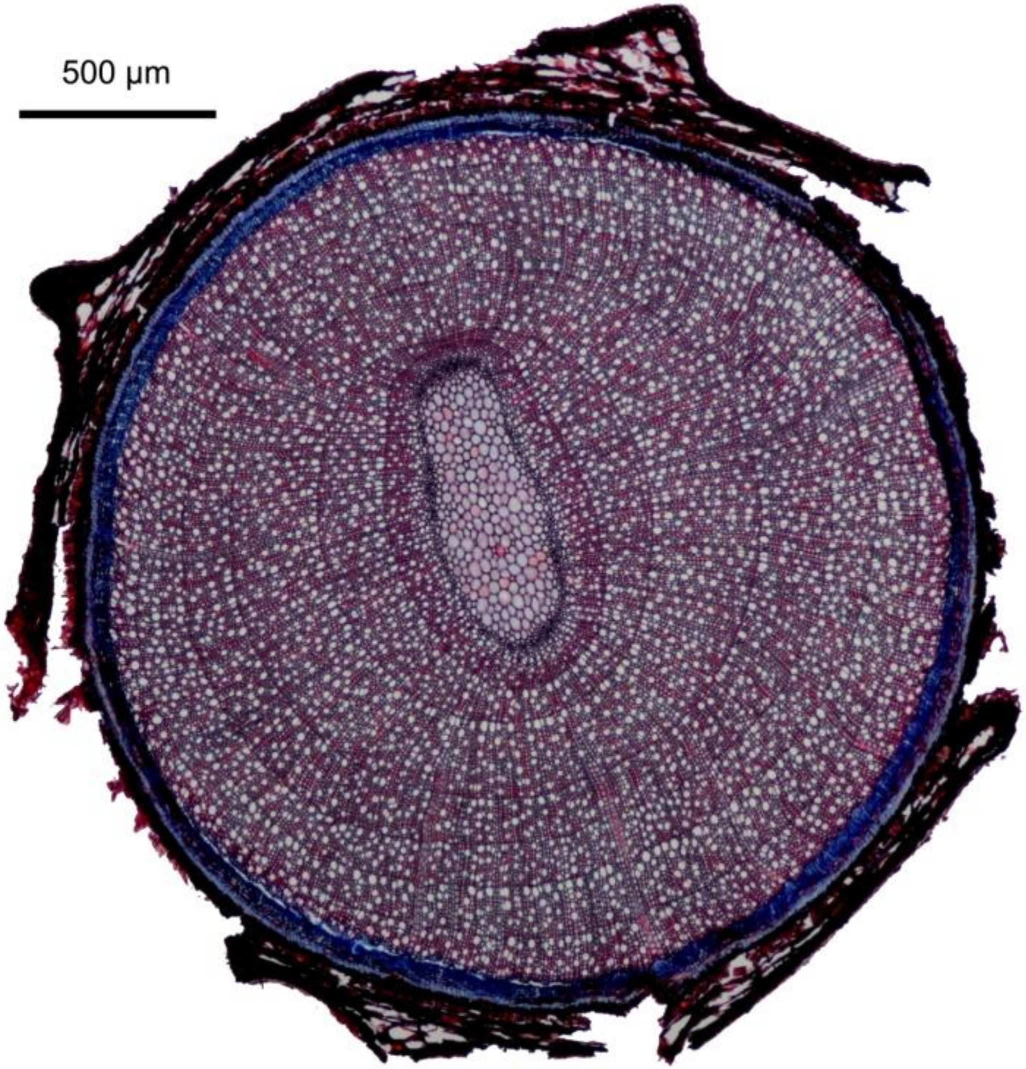

Figure S1. Cross-section (20 µm width) of a 7-year-old *Vaccinium myrtillus* ramet stained with Safranin and Astrablue.
