# Supplementary material for: Growth and Phenology of Three Dwarf Shrub Species in a Six-Year Soil Warming Experiment at the Alpine Treeline
Source: PLoS One. 2014 Jun 23;9(6):e100577. doi: 10.1371/journal.pone.0100577 (PMC4067323; doi:10.1371/journal.pone.0100577)
Supplement: Figure S2 — Age of Vaccinium myrtillus for each soil warming treatment and plot tree species combination. (PDF) [file pone.0100577.s002.pdf]

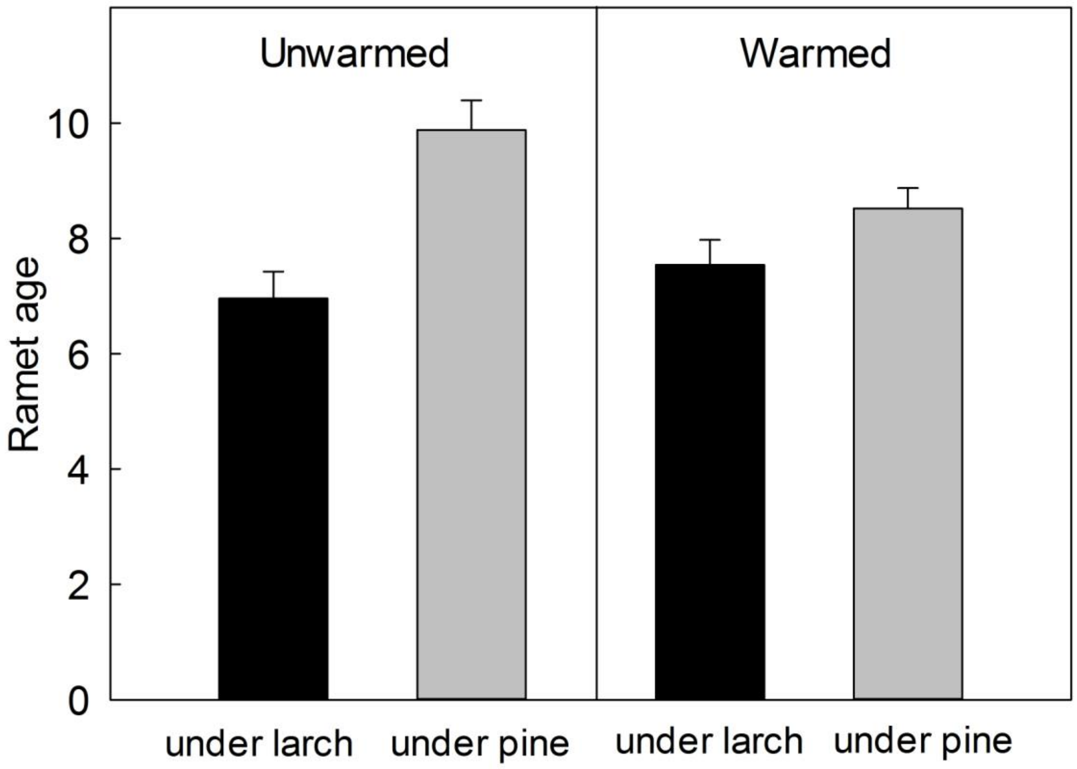

Figure S2. Age (mean +1SE) of *Vaccinium myrtillus* ramets for each soil warming treatment and tree species present in the plot (larch or pine). Significant ($P < 0.05$) age differences were found between ramets in plots with larch and those with pine, and the soil warming x plot tree species interaction was marginally significant ($P < 0.10$).
