# Supplementary material for: Growth and Phenology of Three Dwarf Shrub Species in a Six-Year Soil Warming Experiment at the Alpine Treeline
Source: PLoS One. 2014 Jun 23;9(6):e100577. doi: 10.1371/journal.pone.0100577 (PMC4067323; doi:10.1371/journal.pone.0100577)
Supplement: Table S1 — Climate conditions in the experimental plots during all study years (2007–2012). (PDF) [file pone.0100577.s003.pdf]

Table S1. Climate conditions in the experimental plots during all study years (2007-2012). Mean air temperature and total precipitation at a climate station located *c*. 100 m below the experimental site are given for the main vegetation period, June-August.

Year	Snowmelt date	Air temperature (°C) (mean JJA)	Precipitation (mm) (sum JJA)
2007	07 May	9.5	469
2008	29 May	9.7	470
2009	23 May	10	463
2010	29 May	9.7	539
2011	12 May	9.3	472
2012	28 May	10.7	645
